# The Present State of the Question—On the Glucogenic Function of the Liver

**Published:** 1856-04

**Authors:** 


### ARTICLE VII.

### *The Present State of the Question—On the Glucogenic Function of the Liver.*

In our last quarterly summary of chemistry, we gave an account of the interesting discussion on the subject of the power of the liver to transform albuminous materials so as to generate sugar, and supposed that the decision of the academy had settled the question. Such, however, is not the case. M. Figuier is far from considering himself beaten. He returns to the charge with great vigor, and argues his case with much pertinacity and ingenuity.

He again takes up the question of the presence of sugar in the blood of the *vena porta*, which he failed to show when experimenting before the committee. It will be remembered that he used, on that occasion, Frommhertz's test, (tartrate of copper dissolved in solution of potash,) but that the committee objected to it as unsatisfactory, and when, at their instance, he resorted to the fermentation test, he failed to evolve carbonic acid from the portal blood. The object of his third memoir is to point out the causes of this failure.

He asserts that in the portal blood, there is a peculiar principle which opposes fermentation, and which must be removed before that change can take place. To prove this, he took blood from the portal vein of a large dog, which had been fed several days on horse flesh, treated it with alcohol, acidulated it with acetic acid, evaporated it to dryness, treated the residue with distilled water, and filtered to get rid of albuminous coagula. The liquor was divided into two equal parts, one of which, without further treatment, was mixed with yeast, but failed to ferment. The second portion was boiled two or three minutes with five drops of nitric acid. An albuminous or caseous sediment was deposited, the clear yellow liquid filtered off, and exactly neutralized with carbonate of soda. Then, on being mixed with yeast, it evolved carbonic acid, and after fermentation, responded to the tests for alcohol.

In this way, he says, he has already succeeded in exhibiting the presence of sugar in the portal blood by the fermentation test. He then goes on to show that Bernard's process of "killing the animal and taking the blood in the hepatic veins, that is to say, in the very midst of the saccharine tissue of the liver, is vicious." The rest of his paper is taken up with arguments on physiological principles, with which it is hardly worth while to trouble our readers.

Bernard, in his reply, contradicts the facts, and says that he has repeated Figuier's experiments several times, but has never obtained his results; whereas, on the other hand, he has never failed to get the fermentation, when those circumstances favorable to the presence of sugar in the blood of the portal vein exist. Hence he is disposed to regard the anti-fermentation principle as a figment of his opponent's imagination. Having thus disposed of M. Figuier, he goes on to consider the question how sugar is formed in the liver.

He gives a brief sketch of the various hypotheses to account for the origin of glucose; that of Schmidt, who believes its production to depend upon an oxydation of fatty matter circulating in the blood; that of Lehmann, who thinks it due to the splitting of hæmatosine into sugar and a nitrogenous matter, a change which that eminent chemist has actually accomplished, out of the body, on crystallized hæmatosine; and finally that of Frerichs, who believes the glucogenic action to be due to a power residing in the liver of decomposing certain organic matters which would give rise to urea and sugar. In all these he sees a tacit admission of a certain catalytic force exerted by glandular organs upon the blood in its passage through them. He inclines to another explanation, and thinks the action should be sought in the tissue of the gland itself.

To determine this point he carefully removed the liver of a recently killed dog, passed a powerful stream of water through the portal vein, till the liver became pale, and the water issued from the hepatic veins perfectly colorless. The washing was still continued until the clear fluid gave no indication of sugar by the most delicate tests. The tissue of the liver was also examined and found to be wholly devoid of sugar. The organ was left in a vessel at the common temperature, and in twenty-four hours, was found to be strongly saccharine. This experiment its author considers to establish the facts that the liver contains two substances:

1. Sugar very soluble in water, which is carried away with the blood by washing.

2. Another matter so sparingly soluble in water as to remain fixed in the hepatic tissue, after the latter has been deprived of its sugar by washing for forty minutes, and which is capable of being changed into sugar by a sort of fermentation.

This property is entirely destroyed by cooking; portions of the same liver, remaining free from glucose or containing much of it, according as they were cooked or left raw. Twenty-four hours are sufficient to exhaust this material, since if at the end of that time the liver is washed free from sugar, it is never formed again. Another experiment proved that this substance was also insoluble in alcohol and that the dried hepatic pulp was capable of generating sugar when moistened with water. This matter belongs exclusively to the liver, since there are no traces of it in the portal or any other blood.

During life, this matter being constantly renewed in the tissue by its nutrition, serves as a perennial source of glucose. It follows from these experiments that the liver alone can generate glucose.

Shortly after this, the celebrated author of physiological chemistry, C. G. Lehmann, appeared with another memoir, "on the search for sugar in the blood of the vena porta." In this he states his method of detecting sugar in the blood under dispute. He takes the advantage of the known property possessed by the different varieties of sugar, of forming with potash, a precipitate insoluble in alcohol. He treats the blood with alcohol, evaporates the solution to dryness, and exhausts this extract with highly concentrated alcohol. To this solution, potash dissolved in alcohol is added, and saccharate of potassa falls in a deliquescent mass, very soluble in water. The foreign substances such as chloride of potassium, carbonate of potash, and organic matter, do not interfere with the subsequent test, which is the old one of sulphate of copper and potassa. So delicate is this test that the author has succeeded in obtaining unmistakeable evidence of the presence of sugar in human urine to which one-hundred-thousandth of glucose had previously been added. He also discovered by it, sugar in the ovaries of mammalia, as well as in the albumen of birds eggs.

As an accessory test, he recommends that another portion of the saccharate of potassa be slightly audified with tartaric acid, and treated with yeast to produce fermentation. He has never noticed any substance thrown down with the saccharate which at all interferes with fermentation.

He objects to Figuier's plan of obtaining blood in such large quantities from the portal vein, on the ground, that such copious depletion brings not only blood from the neighboring vessels, but even fluids from the surrounding tissues, in virtue of the law of diffusion. He objects also, that, as the quantity of blood in an adult man does not exceed one-tenth of his weight, on taking such large quantities from the portal vein, it is absolutely certain that other blood must be mingled with it.

He, therefore, first kills the animal subjected to thê experiment, then ties the portal vein close to the liver and empties it of its blood. Thirty-five to eighty grammes have thus been obtained from a single animal. Operating in this manner, this distinguished chemist has always failed to obtain sugar from the portal blood.

Anticipating an objection, which has actually been made to the small amount of blood employed in this investigation, he operated upon three dogs fed with meat, collecting and uniting the blood from all three. This amounted to 217½ grammes of blood, (equal to 3350 grains, or nearly half a pound avoirdupois.) Notwithstanding this large quantity was employed, no trace of sugar was found.

In order to determine whether glucose could be found when a large quantity of blood was taken from the portal vein of a living dog, in which event, according to the preceding observations, it must necessarily have come from other sources besides the normal portal blood, he drew quantities approximating those on which M. Figuier operated. In the experiments of this kind, he obtained glucose every time.

His conclusions from these experiments he states to be:

"1. When too much blood is drawn from the *vena porta*, we do not obtain suitable blood, nor such as circulates normally in the vessel during life.

"2. When we place ourselves in such conditions as to obtain only the pure blood of the *vena porta*, no glucose is found in it during the digestion of meat."

The next question which he set himself to solve, was that of the presence of a glucoside or copulated sugar in the blood of the portal vein. To determine this, he digested the alcoholic extract of portal blood with diastase and then boiled it with a few drops of sulphuric or nitric acid. No fermentable substance was at any time obtained. The examination of the stomach and small intestines of dogs fed on meat, for the same substance, led also to negative conclusions.

As for the statement of Figuier, that the portal blood contains a substance which prevents alcoholic fermentation, Lehmann unites with Bernard in denying it. He added sugar in small quantities to the alcoholic extract of this blood, and never found the alcoholic fermentation to be prevented or retarded. Furthermore, he thinks that, if these experiments of Figuier were correct, they would not prove the existence of a substance preventing fermentation, but only the presence of a glucoside or copulated sugar, susceptible of development into glucose in the liver, and would, therefore, confirm instead of controverting Bernard's views.

Figuier, in his reply, disingenuously, as it appears to us, avoids Lehmann's strong objection to his method of obtaining portal blood, and complains that that chemist ought to have employed the same process which he used. In criticising the process of the German chemist, he strongly objects to the use of caustic alkali, which he says forms, with great difficulty, a glucosate with the secondary sugars, and that such salts when formed, are destroyed almost immediately upon coming in contact with water. He further insists that the saccharine ingredient of portal blood cannot be compared chemically with glucose.

In reply to Lehmann's assertion that the discovery of a glucoside would support Bernard's theory, he objects that, in that case, the liver would not be said to secrete sugar, but merely to modify the glucoside *in transitu*, just as the stomach transforms aliment into peptones.

To this point has the interesting discussion under consideration arrived. It must be acknowledged that Figuier has contended with distinguished ability against his great opponents, but it must also be admitted that at present the weight of argument inclines to the other side. How future researches may affect the question it is impossible to foresee. One thing, however, is very certain; we shall emerge from this contest with clearer notions of the necessary precautions in dealing with organic chemistry, and a better idea of all the functions in debate, than we could possibly have had without this searching and acute criticism.

A paper bearing on this subject, from the pen of Dr. Pavy, has been recently published in the proceedings of the Royal Society. He finds that sugar exists in the arteries, the capillaries, and even in the veins, where it is still undergoing destruction, but that this destruction is more complete in some veins than in others, so that while the portal blood may be wholly destitute of sugar, that of the femoral or jugular veins still contains it in appreciable quantity. A moment's consideration of the preceding remarks will show how important a bearing this fact has upon the whole discussion relative to the portal blood.

This led Dr. Pavy to a consideration of the circumstances under which the saccharine matter of the blood is destroyed in the economy. He found that Liebig's hypothesis of its combustion in the lung is not sufficient, for, on obstructing the respiration of an animal nearly to the point of fatal asphyxia, the destruction of sugar still went on. On injecting the blood of one animal through the inflated lung of another, he found the destruction of the sugar to be as great as during life, but when he defibrinated the blood, the change did not occur.

Observations on the disappearance of sugar, in blood out of the body, corresponded with this result. The blood set aside to undergo spontaneous coagulation, was found to contain very different proportions of sugar in its two constituent parts. The serum contained quantities of it, while none could be discovered by the most delicate tests in the coagulum, showing that the organic decomposition is the true cause of the change. The oxygen of the air seems to be concerned in it only so far as it promotes this nitrogenous decomposition. On the completion of the metamorphosis, the blood is found to be acid, a reaction believed to be due to the presence of lactic acid.

Applying these facts, we find that a nitrogenous substance, in a state of change, is capable of exciting a molecular movement in the sugar, which results in the formation of lactic acid. "If the molecular changes, occurring during the decomposition of a nitrogenous substance, be capable of converting sugar into lactic acid, why should not the molecular changes occurring during the building up or elaboration of this same nitrogenized compound effect the same? Indeed, we have seen that the process of destruction is carried on to a certain extent in the systemic capillaries, and more especially in those of the chylopoietic viscera, where the molecular changes of nutrition are also correspondingly carried on with greater activity than elsewhere. So that analogy and experiment would tend to show that the physiological destruction of sugar is owing to a process similar to fermentation induced by the molecular changes occurring in the nitrogenized constituents of the animal during life; and, in accordance with this, we find lactic acid present in the system, and largely separated from arterial blood by the muscular tissue, and the secerning follicles of the stomach."

